# Dimensionality of Motion and Binding Valency Govern Receptor–Ligand Kinetics As Revealed by Agent-Based Modeling

**DOI:** 10.3389/fimmu.2017.01692

**Published:** 2017-11-30

**Authors:** Teresa Lehnert, Marc Thilo Figge

**Affiliations:** ^1^Research Group Applied Systems Biology, Leibniz Institute of Natural Product Research and Infection Biology – Hans Knöll Institute (HKI), Jena, Germany; ^2^Center for Sepsis Control and Care (CSCC), Jena University Hospital, Jena, Germany; ^3^Faculty of Biology and Pharmacy, Friedrich Schiller University Jena, Jena, Germany

**Keywords:** agent-based model, ordinary differential equations, antibody–antigen binding, receptor–ligand interaction, dimensionality of motion, binding valency

## Abstract

Mathematical modeling and computer simulations have become an integral part of modern biological research. The strength of theoretical approaches is in the simplification of complex biological systems. We here consider the general problem of receptor–ligand binding in the context of antibody–antigen binding. On the one hand, we establish a quantitative mapping between macroscopic binding rates of a deterministic differential equation model and their microscopic equivalents as obtained from simulating the spatiotemporal binding kinetics by stochastic agent-based models. On the other hand, we investigate the impact of various properties of B cell-derived receptors—such as their dimensionality of motion, morphology, and binding valency—on the receptor–ligand binding kinetics. To this end, we implemented an algorithm that simulates antigen binding by B cell-derived receptors with a Y-shaped morphology that can move in different dimensionalities, i.e., either as membrane-anchored receptors or as soluble receptors. The mapping of the macroscopic and microscopic binding rates allowed us to quantitatively compare different agent-based model variants for the different types of B cell-derived receptors. Our results indicate that the dimensionality of motion governs the binding kinetics and that this predominant impact is quantitatively compensated by the bivalency of these receptors.

## 1 Introduction

In recent decades, computational biology has developed into an autonomous scientific discipline that has become indispensable for contemporary biological research. Major contributions of computational biology comprise: (i) directing studies by providing insights that cannot otherwise be obtained in wet-lab experiments, (ii) advancing biological research toward a quantitative science through large-scale computations, and (iii) generating experimentally testable hypotheses through simulations of mathematical models.

The strength of mathematical modeling is actually in the simplification of complex processes by focusing on the most relevant aspects of a system. The art of modeling is in the appropriate choice of a mathematical approach that describes all existing experimental data and still can make relevant predictions. At this point a reasonable compromise has to be made between the level of system complexity that is transferred into the mathematical model and the feasibility of simulations with regard to computational resources.

Models based on ordinary differential equations (ODE) are presumably most frequently applied in biological research, even though this modeling approach is only valid if the system under consideration consists of large amounts of constituents, e.g., molecules, that are homogeneously distributed or well stirred in some spatial environment (1). This is because ODE models do not explicitly account for any spatial aspects of a system and changes in system variables, e.g., concentrations of molecules, are consequently described by functions of time that are continuous and deterministic. However, these assumptions, which may be typically appropriate for chemical systems, are for biological systems at best applicable from a macroscopic point of view. In these macroscopic models the biological processes are characterized by two specific types of parameters, which are referred to as *rates* or *reaction rates*. Rates characterize unimolecular processes that occur spontaneously and have unit 1/time. Reactions involving two types of molecules, i.e., bimolecular processes, are characterized by reaction rates with unit 1/(concentration × time). Typical experimental assays to determine these macroscopic rates for uni- and bimolecular processes are the adhesion frequency assay and the surface plasmon resonance assay (2). The advantage of ODE models is that they are based on a minimal set of parameters and can be formulated with relative ease (1, 3), which makes them belonging to the so-called simple modeling approaches (4). Deterministic ODE models may be extended to account for the stochasticity of chemical reactions in solution. Various numerical schemes have been introduced by Gillespie to sample the underlying master equation for the probability to find the system in a particular state at a given time (5). These are referred to as the *direct method* (6) and the *first reaction method* (7) and were later advanced for computational speed-up with the *next reaction method* by Gibson and Bruck (8). Albeit more detailed than deterministic ODE models, all these approaches have in common that a macroscopic viewpoint on the system is taken.

In contrast, agent-based models (ABMs), which belong to the so-called detailed modeling approaches (4), consider biological systems from a microscopic viewpoint by taking details of their individual constituents in space and time into account. A system’s constituents, e.g., molecules and/or cells, are represented by agents in the model and their motion in a specific spatial environment as well as their stochastic interactions with other agents are monitored in the simulations. In this microscopic modeling approach, all reactions are performed with a specific probability per time-step. This implies that not only the rates for unimolecular processes are measured in unit 1/time, but also the reaction rates for bimolecular processes, because the microscopic reactions are between two single molecules and not between concentrations of molecules as is the case for macroscopic ODE models. The microscopic rates for molecular interactions could be experimentally measured using thermal fluctuation assays (2). However, the level of detail represented by ABM comes at the price of a relatively large number of model parameters, which may be unknown and/or even inaccessible to experiment (1, 9), and simulations of ABM are typically associated with a high computational load (10, 11).

In this study, we focus on specific receptor–ligand (RL) binding, i.e., antibody–antigen binding as a central part of the adaptive immune response, and model this process in a comparative fashion by ODE models and by ABM. Binding between receptors and ligands represents an essential process in the immune system by which important information is transferred. For example, in the process termed *opsonization*, pathogen-derived antigens can be neutralized and labeled by antibodies for removal from the organism. Antibodies are soluble molecules that play a key role in the humoral response of adaptive immunity (12), because they can bind antigens with high affinity and can provide life-long protection against specific antigens. Of interest, antibodies do also exist as membrane-anchored molecules on B lymphocytes and are then referred to as B cell receptors (BCR). Binding of cognate antigen by BCR activates naïve B cells in lymphoid organs, such as spleen and lymph node (13), and this may initiate a germinal center (GC) reaction for antibody affinity maturation (12). During a GC reaction, B cells are proliferating and mutating their BCR followed by the selection of B cells with BCR that have high affinities to presented antigens. B cells with BCR that successfully accomplished the selection procedure differentiate into plasma cells that produce large amounts of these BCR as soluble antibodies. The GC reaction has been the subject of various interdisciplinary studies combining experimental and theoretical investigations (5, 14–17). In particular, it could be shown that the GC reaction is not only initiated by antigen binding to BCR on B cells, but that its termination is as well regulated by the high-affinity antibodies produced in soluble form (18). Taken together, antibodies represent a prime example for this study because of three reasons: (i) they exist as soluble as well as membrane-anchored receptors, (ii) they have a peculiar Y-shaped morphology that raises the question on its impact on RL binding as compared to spherically shaped receptors, and (iii) they have two binding sites and can bind antigen mono- or bivalently. The computational biology approach that is pursued in this study allows investigating the relative importance of receptor morphology, binding valency and dimensionality of motion that depends on receptors being soluble or membrane anchored on a cell. Applying different modeling approaches, e.g., ODE models and ABM, in a comparative fashion enables a quantitative mapping of the macroscopic and microscopic viewpoint on RL binding dynamics.

## 2 Materials and Methods

### 2.1 Microscopic Modeling of Receptor–Ligand Binding

**Figure 1:**
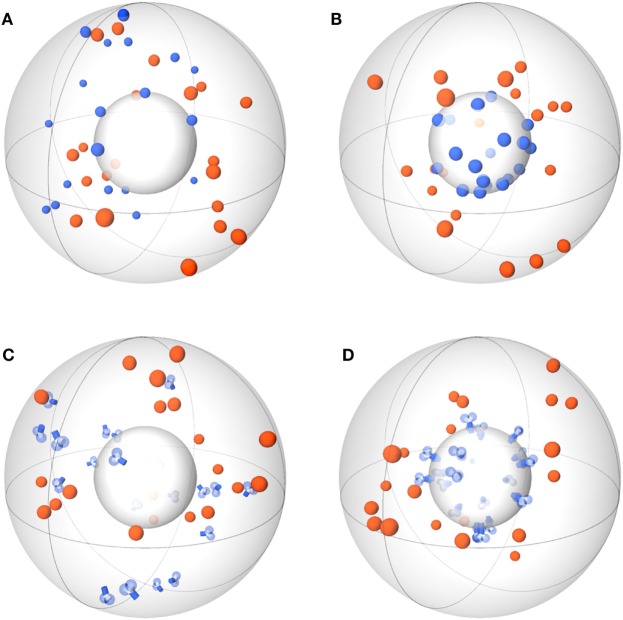
Schemes of ABM variants for receptor–ligand binding. The ABM variants are composed of the same spherical environment (large gray sphere) containing a spherical cell (small gray sphere) at the center. Ligands (orange) are always soluble, whereas receptors (blue) are studied in the variants: spherical receptor morphology in **(A)** soluble (O-SOL) or **(B)** membrane-anchored (O-MEM) form and Y-shaped receptor morphology in **(C)** soluble (Y-SOL) or **(D)** membrane-anchored (Y-MEM) form.

**Figure 2:**
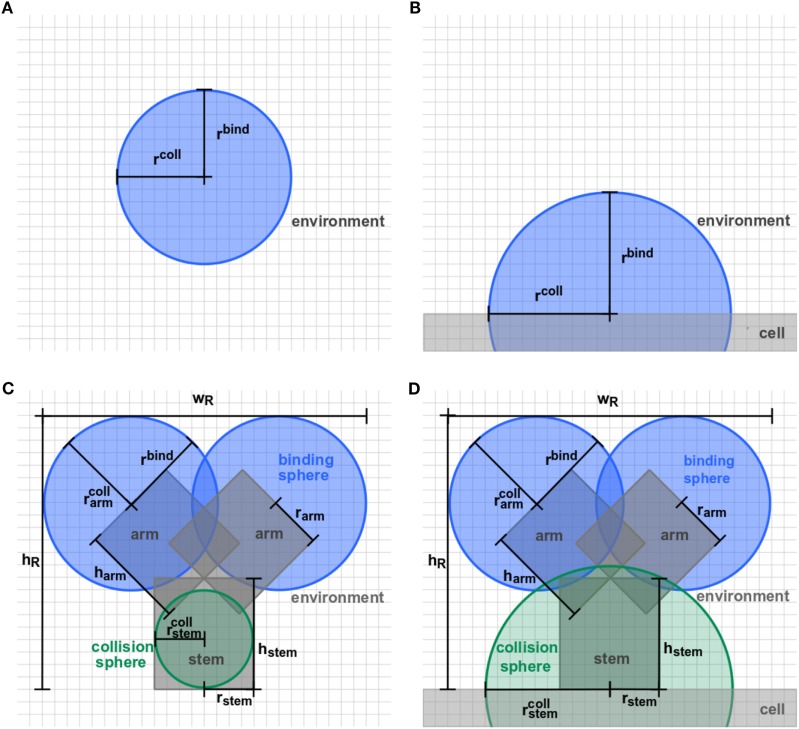
Detailed representation of receptor morphologies. Two-dimensional projection of three-dimensional receptors in ABM variants **(A)** O-SOL, **(B)** O-MEM, **(C)** Y-SOL, and **(D)** Y-MEM. Each receptor consists of binding spheres and collision spheres that may be overlapping in position and size. Ligands can bind by encountering a receptor’s binding sphere but are prohibited to penetrate receptors by the collision spheres. Details on the parameter values are provided in Table S1 in Supplementary Material.

Agent-based models (ABMs) are widely used in computational biology to simulate processes at the microscopic scale (9–11, 19). The individual constituents of the biological system under consideration are represented as agents that can move in a defined spatial environment and can interact with each other according to specific rules. We studied receptor–ligand (RL) binding and, in particular, the impact of specific receptor properties on the dynamics of the binding process. While ligands were modeled as molecules in solution with spherical shape, we considered receptors with different morphologies, i.e., being either spherically shaped (O) or Y-shaped (Y), and in settings with different dimensionality of motion, i.e., receptors in solution (SOL) or membrane anchored (MEM) on the surface of a cell. The four combinations of receptor properties are depicted in Figures 1 and 2, and give rise to four different ABM variants. These are denoted by their receptor properties, respectively, as O-SOL (see Figures 1A and 2A), O-MEM (see Figures 1B and 2B), Y-SOL (see Figures 1C and 2C), and Y-MEM (see Figures 1D and 2D). Simulations of the different ABM variants are shown in Videos S1–S5 in Supplementary Material. While in what follows we describe the general setup of the ABM, a detailed overview of the model parameters and of their corresponding values is provided in the Table S1 in Supplementary Material.

#### 2.1.1 Model System

In this study, we considered the model system of a B cell with Y-shaped B cell receptors (BCR), because these receptors do as well exist in a soluble form as antibodies. In the ABM, BCR with their *Fab*-fragments as binding sites are represented by a cylindrical stem with two cylindrical arms and spherical binding regions at the distal sides, which are hereafter referred to as *binding spheres*. A schematic representation of the BCR in soluble and membrane-anchored form is shown, respectively, in Figure 2C for ABM variant Y-SOL and in Figure 2D for ABM variant Y-MEM. The binding spheres on top of each arm represent the active binding sites of the BCR, whose surface areas are estimated from the size of *Fv*-regions, i.e., the variable parts of the BCR *Fab*-arms. Thus, the binding spheres implicitly account for the attractive short-range interactions between the binding sites of receptors and ligands (20–23). For the reason of comparison between BCR and spherically shaped receptors, we set the values of binding radii such that the effective area of all binding spheres are of comparable size, as can be inferred from the relative receptor sizes in Figures 2A,B for ABM variants O-SOL and O-MEM, respectively. For the same reason, when comparing Y-shaped and spherically shaped receptors, we impose the condition that receptors can only bind one ligand at a time. In addition, we also compared Y-shaped receptors that can bind mono- and bivalently.

#### 2.1.2 Molecular Diffusion and Interaction

Receptors and ligands perform diffusive motion in the ABM. The corresponding diffusion coefficients can vary by orders of magnitude for soluble and membrane-anchored receptors. Diffusion coefficients were estimated based on the Stokes-Einstein equation (24) and the values for the corresponding ABM variants (see Table S1 in Supplementary Material) were calculated as outlined in Supplementary Material. In this study, we aim to investigate the impact of the dimensionality of motion for different receptor morphologies during the process of RL binding. In the ABM, molecules with diffusion coefficient *D* move per time step Δ*t* the specific distance $\Delta s=\sqrt{2dD\Delta t}$ in a direction of the *d*-dimensional space that is chosen from a uniformly random distribution. This motion involves also a random rotation of Y-shaped receptors around their two axes in a spherically uniform fashion.

Two types of interaction processes are possible in the ABM: binding of receptor and ligand to form a molecular complex and dissociation of such a complex into individual receptor and ligand. The latter process occurs with rate $k_{off}^{micro}$ and translates into the probability $p_{off}^{micro}=k_{off}^{micro}\Delta t$ that a complex dissociates during one time step Δ*t*. In this study, we set the microscopic and macroscopic dissociation rates to be equal, i.e., $k_{off}^{micro}=k_{off}^{macro}$. As analyzed in detail in Supplementary Material, this approach is valid for typical parameter values of antibody–antigen dissociation rates, implying that dissociation and rebinding are relatively rare processes. On the other hand, binding of diffusing receptor and ligand requires that these molecules first encounter each other in the spatial environment. Then, upon contact of the ligand with the respective binding sphere of a receptor, binding occurs with probability $p_{on}^{micro}=k_{on}^{micro}\Delta t$, where $k_{on}^{micro}$ denotes the microscopic binding rate with unit s^−1^. Note, that this rate is conceptually different from the macroscopic reaction rate $k_{on}^{macro}$ with unit µm^3^ s^−1^, because the latter incorporates the process of encounter of molecules in a spatially homogeneous system by their concentrations. In this study, we establish a relation between $k_{on}^{micro}$ and $k_{on}^{macro}$ by mapping the microscopic and macroscopic RL binding kinetics onto each other.

#### 2.1.3 Implementation and Simulation

We implemented the ABM in a spherical environment with the cell positioned at its center and for reasons of comparison this was the same in all four ABM variants. The boundary condition at the outer boundary of the environment was chosen to be random-periodic for molecule motion, i.e., a molecule leaving the system at one point was entering the system at another random position of this boundary, where the newly added molecule was given an entirely new identity. At the inner boundary of the cell surface, reflecting boundary conditions were imposed. By applying these realistic boundary conditions, we ensure that the number of molecules in the system is constant during the simulation time.

For a highly realistic implementation of RL binding dynamics, a continuous space representation was used and combined with the neighbor-list method (25, 26) to speed up the detection of interaction partners in this off-lattice approach. Molecules in motion may approach each other and become overlapping. We implemented a push-back procedure, such that the overlap by the moving molecule was reduced to a point contact with the other molecule. Thus, we imposed the condition that molecules cannot penetrate each other and this choice impacts on the effective reaction volume between the molecules.

For reasons of comparison between the different ABM variants, we use the same time step Δ*t* in each simulation, such that changes in the simulation results can be clearly attributed to differences in the receptor morphology, the dimensionality of motion and/or binding valency. To this end, we determine the time step

(1) $$\Delta t=\text{min}\;\left(\text{min}\;\left({k_{off}^{micro}}^{-1},{k_{on}^{micro}}^{-1}\right),\;\text{min}\left(\Delta t_{R},\Delta t_{L}\right)\right),$$

from the smallest considered rate of binding $\left(k_{on}^{micro}\right)$ and dissociation $\left(k_{off}^{micro}\right)$ as well as the smallest time step associated with a diffusion step in space that does not exceed the radius of receptors (Δ*s_R_*) and ligands (Δ*s_L_*). The time steps of receptors (Δ*t_R_*) and ligands (Δ*t_L_*) are given by

(2) $$\Delta t_{R,L}\;=\frac{\Delta s_{R,L}^{2}}{2d\;D_{R,L}}\;.$$

**Figure 3:**
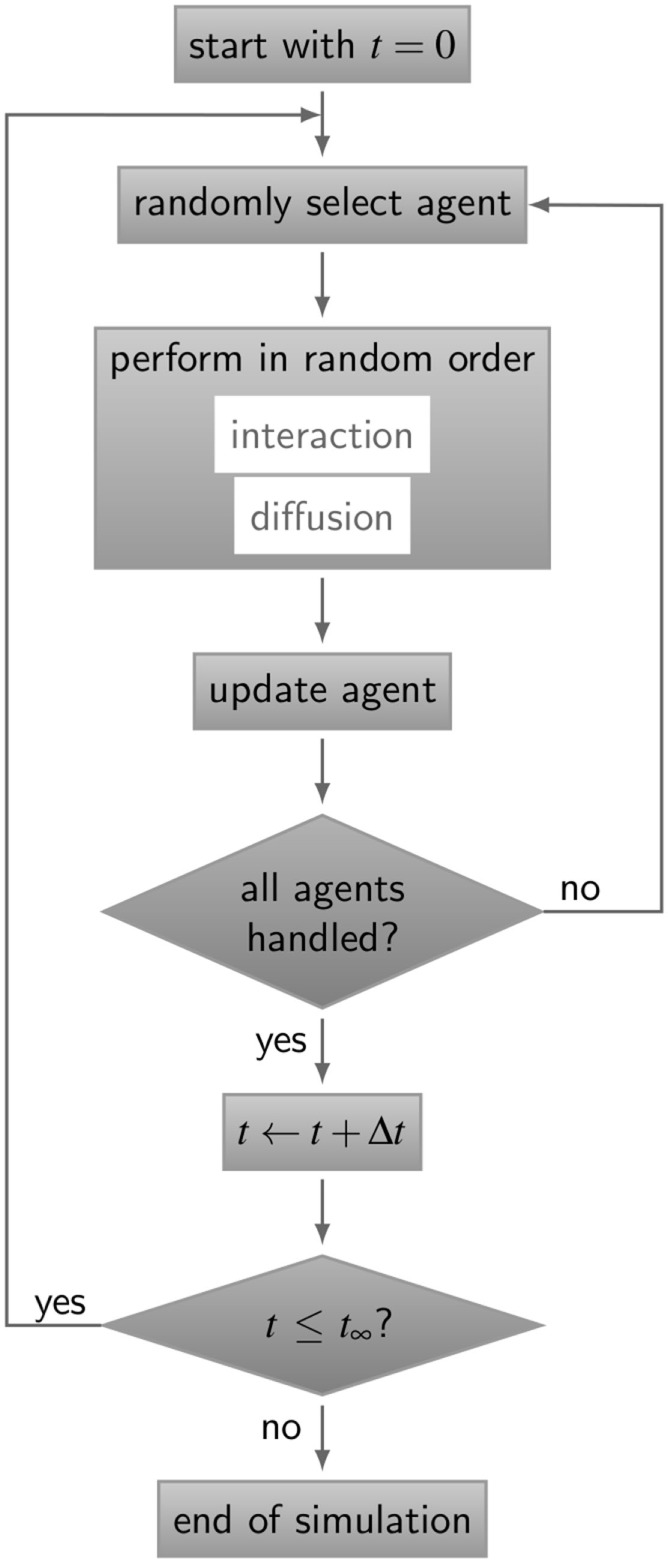
Flow chart of the ABM simulation algorithm for receptor–ligand binding kinetics. The gray boxes represent operations and are connected by directive arrows depicting the sequence of the ABM simulation algorithm. In each time step Δ*t*, all agents perform diffusive motion and undergo interactions with other agents in random order until simulation time *t_∞_*is reached. Simulations of all ABM variants are shown in Videos S1–S5 in Supplementary Material.

The simulation algorithm for RL binding dynamics is based on random selection dynamics (5). Each molecule is updated per time step with regard to its diffusion and interaction that are performed in random order applying the acceptance-rejection method (27). A flowchart of the algorithm is shown in Figure 3. For the model system under consideration, i.e., a B cell with a number of BCR in the order 10^5^ and an equal amount of ligands, simulation run times would exceed all limits. In fact, it can be estimated that the ratio of the typical simulation time over the simulated real time becomes as large as 10^9^. Therefore, since the size of the time step is determined by the accurate resolution of molecular motion and interaction, we down-scale the number of molecules and decrease the system size while keeping the molecular concentration constant. The details of the down-scaling procedure are described in Supplementary Material and the associated values are summarized in Table S2 in Supplementary Material. All simulations were performed after down-scaling the number of molecules by a factor *s* = 10^−2^, i.e., reducing the B cell size by a factor 10 and the number of BCR to the order 10^3^.

The ABM framework was implemented in the object-oriented programming language C + +.

### 2.2 Macroscopic Modeling of Receptor–Ligand Binding

Modeling RL binding from a macroscopic point of view can be done in a straightforward fashion using ordinary differential equations (ODE). This approach is appropriate to describe chemical processes where reaction partners occur in large amounts and are homogeneously distributed in the spatial environment. Consequently, ODE models represent time-dependent changes of molecule concentrations in a continuous and deterministic fashion. We considered the binding of receptors (*R*) and ligands (*L*) to form a molecular complex (*C*) as well as their unbinding:

(3) $$R+L\underset{k_{off}^{macro}}{\overset{k_{on}^{macro}}{⇄}}\;C.$$

Here, $k_{on}^{macro}$ is the reaction rate for binding, $k_{off}^{macro}$ is the dissociation rate and the corresponding association constant *K_a_* is defined by their ratio: $K_{a}=k_{on}^{macro}∕k_{off}^{macro}$.

The reaction equation (3) was then translated into the coupled system of ODE:

(4) $$\frac{dR}{dt}=-k_{on}^{macro}RL+k_{off}^{macro}C,$$

(5) $$\frac{dL}{dt}=-k_{on}^{macro}RL+k_{off}^{macro}C,$$

(6) $$\frac{dC}{dt}=+k_{on}^{macro}RL-k_{off}^{macro}C.$$

Assuming that initially no molecular complexes exist, *C* (*t* = 0) = 0, it follows from the relations *R*(*t*) = *R*(0) – *C*(*t*) and *L*(*t*) = *L*(0) – *C*(*t*) that it is sufficient to solve the non-linear equation for *C*(*t*):

(7) $$\frac{dC}{dt}=\alpha C^{2}-\beta C+\gamma ,$$

where we defined the constants

(8) $$\alpha =k_{on}^{macro},$$

(9) $$\beta =k_{off}^{macro}+k_{on}^{macro}\;\left[R\left(0\right)+L\left(0\right)\right],$$

(10) $$\gamma =k_{on}^{macro}\;R\left(0\right)L\left(0\right).$$

The ODE for *C*(*t*) can be solved by the separation of variables and yields the analytical solution:

(11) $$C\left(t\right)=C_{-}\;C_{+}\;\frac{1-\;e^{\alpha \left(C_{+}-C_{-}\right)t}}{C_{-}-\;C_{+}\;e^{\alpha \left(C_{+}-C_{-}\right)t}}$$

with

(12) $$C_{\pm }=\frac{\beta }{2\alpha }\pm \sqrt{\frac{\beta^{2}}{4\alpha^{2}}-\frac{\gamma }{\alpha }}\;.$$

Note that the concentration *C*(*t*) is associated with the number of receptor–ligand (RL) complexes in the microscopic model (see Materials and Methods section 2.1.2).

### 2.3 Mapping Microscopic and Macroscopic Binding Kinetics

A relation between the macroscopic and microscopic viewpoint on the binding kinetics of receptors and ligands can be established *via* the corresponding reaction rates for RL binding $k_{on}^{macro}$ and $k_{on}^{micro}$. Given the concentration of molecular complexes C(t) (see equation (11)), we fit this analytical solution from macroscopic binding kinetics to the numerical results of simulations obtained from ABM at the microscopic level. This yields the desired relation $k_{on}^{macro}\left(k_{on}^{micro}\right)$ that can be compared for different ABM variants.

The fitting procedure was performed within the open source programming language R (28). We used the function *nls*() that returns optimal parameter values of non-linear model equations by least-squares fitting. In particular, we used the fitting algorithm option “port” that refers to the adaptive non-linear least-squares algorithm NL2SOL (29) provided by the Port library. The algorithm adaptively switches between the Gauss-Newton method and an augmented Hessian approximation (30).

In practice, we applied the fitting procedure in two different respects: (i) The macroscopic binding rate $k_{on}^{macro}$ in equation (11) was estimated from fitting to the data points obtained from numerical simulations with the ABM over time. (ii) The values determined for $k_{on}^{macro}$ were used as data points to fit the optimal parameter values of the Hill equation $k_{on}^{macro}\left(k_{on}^{micro}\right)$ (see equation (13)) in order to map the microscopic and macroscopic binding kinetics.

## 3 Results

In this section, we present our simulation results on receptor–ligand (RL) binding by comparing the dynamics of individual receptors and ligands at the microscopic level with the population kinetics at the macroscopic level. The population kinetics can be straightforwardly described by a coupled system of ordinary differential equations (ODE), whereas agent-based models (ABM) resolve spatial structures of receptors and ligands and account for the dimensionality of the spatial environment in which these molecules diffuse and interact. In particular, we study monovalent receptors with different morphologies, i.e., being either spherically shaped (O) or Y-shaped (Y), and in settings with different dimensionality of motion, i.e., in solution (SOL) or membrane anchored (MEM). While ligands are throughout considered as being in solution and as having spherical shape, the four combinations of receptor properties give rise to four different ABM variants that are denoted by their receptor properties, respectively, as O-SOL, O-MEM, Y-SOL, and Y-MEM. These are schematically depicted in Figure 1 and the differences between receptors are shown in Figure 2. In addition, videos of simulations for the different ABM variants with monovalent receptors are provided in Videos S1–S5 in Supplementary Material, where Videos S1-S4 represent down-scaled systems with factor *s* = 10^−2^, while Video S5 shows a simulation of ABM variant Y-MEM with *s* = 1. A flow chart of the simulation algorithm is provided in Figure 3 and details on the implementation of the ABM and on the model parameters are given in the Materials and Methods section.

### 3.1 Binding Kinetics for Different Receptor Properties Qualitatively Comparable

**Figure 4:**
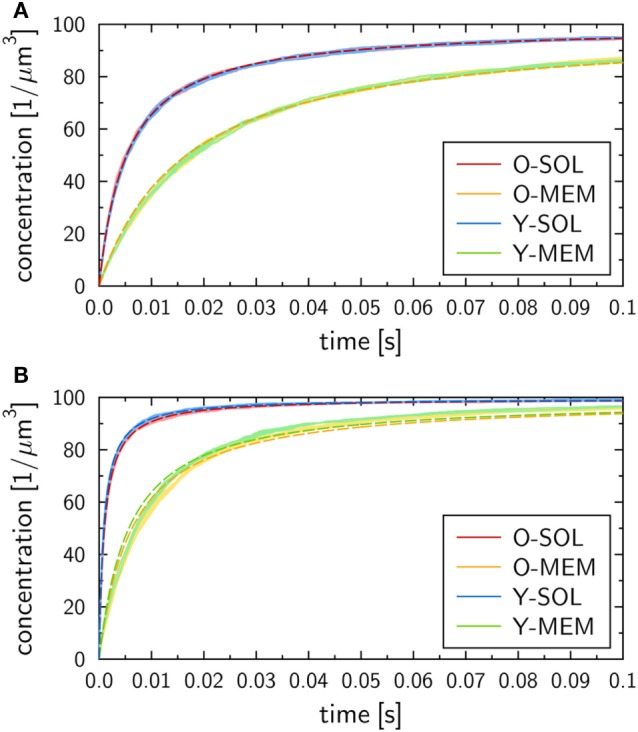
Receptor–ligand binding kinetics for the four ABM variants. Time-dependent concentration of RL complexes with monovalent receptors as obtained from simulations of all four ABM variants with dissociation rate $k_{off}^{micro}=0.1\;{\text{s}}^{-1}$ and binding rate **(A)** $k_{on}^{micro}=10^{6}\;{\text{s}}^{-1}$ or **(B)** $k_{on}^{micro}=10^{7}\;{\text{s}}^{-1}$. Dark and pale lines in different colors represent, respectively, mean values and standard deviations of five simulation runs per ABM variant. Dashed lines indicate the corresponding ODE models after fitting the macroscopic reaction rate $k_{on}^{macro}$.

The binding kinetics at the macroscopic level, which can be determined from the analytical solution of the ODE model (see Materials and Methods section), was observed to be in qualitative agreement with the simulation results of all four ABM variants with monovalent receptors at the microscopic level. This can be seen from the ABM simulation results in Figure 4, where the microscopic rate for RL dissociation was fixed at $k_{off}^{micro}=0.1\;{\text{s}}^{-1}$, while the microscopic rate for RL binding was set to $k_{on}^{micro}=10^{6}\;{\text{s}}^{-1}$ (Figure 4A) and $k_{on}^{micro}=10^{7}\;{\text{s}}^{-1}$ (Figure 4B). Note that we provide the concentration of molecular complexes in units 1/µ m^3^ to enable the comparison of the binding dynamics simulated by ODE and ABM variants with soluble and membrane-anchored receptors. Since the initial numbers of receptors and ligands as well as the system volumes are identical in all models and simulations, we basically perform a comparison with regard to the number of complexes in each system. In general, we observed that the impact of the stochasticity on RL binding dynamics in the ABM is small, e.g., the relative standard deviation in the number of RL complexes was found to be around 1% for equilibrated systems (see the thickness of curves in pale colors in Figure 4). This is due to the large number of molecules in each simulation, such that five repetitions—involving in total the simulation of 10^4^ molecules—yielded vanishingly small standard deviations.

We generally found a decrease in the concentration of free receptors and ligands with time, which was naturally associated with an increase in the concentration of RL complexes. This observation was robust against variations in the receptor properties, i.e., all four ABM variants—O-SOL, O-MEM, Y-SOL, and Y-MEM—showed the same qualitative behavior. Thus, the qualitative agreement with the macroscopic binding kinetics based on the ODE was not limited to the ABM variant O-SOL as its direct microscopic counterpart. Therefore, in what follows, the analytical ODE solution can be used to fit the simulation results of all four ABM variants and to characterize them by their quantitative differences in the macroscopic binding rate $k_{on}^{macro}$. Note that this is the only free model parameter, since the dissociation of RL complexes occurs spontaneously at both the microscopic and macroscopic level implying that the corresponding rates are identical: $k_{off}^{macro}=k_{off}^{micro}$. Arguments for this relation between macroscopic and microscopic dissociation rates are provided based on the analysis in Supplementary Material.

### 3.2 Receptor Properties Have Quantitative Impact on Binding Kinetics

At the quantitative level, we observed differences in the binding kinetics depending on the receptor properties as well as on the microscopic binding rate $k_{on}^{micro}$. As could be expected, formation of RL complexes occurred slower for smaller $k_{on}^{micro}=10^{6}\;{\text{s}}^{-1}$ (Figure 4A) than for larger $k_{on}^{micro}=10^{7}\;{\text{s}}^{-1}$ (Figure 4B). Moreover, for a fixed value $k_{on}^{micro}$, the ABM variants with monovalent receptors in solution—O-SOL (red lines) and Y-SOL (blue lines)—exhibited quantitative agreement in the binding kinetics. While for the corresponding ABM variants with membrane-anchored receptors—O-MEM (orange lines) and Y-MEM (green lines)—this quantitative agreement was also observed, a quantitative difference in the binding kinetics between receptors in solution and membrane-anchored receptors was clearly visible (see Figure 4).

Using the analytical ODE solution of the binding kinetics, we fitted the simulation results of all four ABM variants to characterize them by their quantitative differences in the macroscopic binding rate $k_{on}^{macro}$. The fitted curves are shown in Figure 4 and yielded for $k_{on}^{micro}=10^{6}\;{\text{s}}^{-1}$ (Figure 4A) the values $k_{on}^{macro}\approx 1.9\;µ{\text{m}}^{3\;\;}{\text{s}}^{-1}$ for the ABM variants O-SOL and Y-SOL and $k_{on}^{macro}\approx 0.6\;µ{\text{m}}^{3}\;{\text{s}}^{-1}$ for the ABM variants O-MEM and Y-MEM. For $k_{on}^{micro}=10^{7}\;{\text{s}}^{-1}$ (Figure 4B), we obtained the values $k_{on}^{macro}\approx 10.5\;µ{\text{m}}^{3}\;{\text{s}}^{-1}$ for the ABM variants O-SOL and Y-SOL and $k_{on}^{macro}\approx 1.7\;µ{\text{m}}^{3}\;{\text{s}}^{-1}$ for the ABM variants O-MEM and Y-MEM. It should be noted that the goodness of the fit, which was evaluated by the error of least squares fitting, was comparable for all simulations with microscopic binding rates in the range $10^{4}\;{\text{s}}^{-1}\leq k_{on}^{micro}\leq 10^{6}\;{\text{s}}^{-1}$. Even though for $k_{on}^{micro}>10^{6}$ the error of least squares fitting for ABM variants with membrane-anchored receptors can be up to two orders of magnitude larger than for those with receptors in solution (see Figure S1 in Supplementary Material), all fitted curves still represented a fair representation of the simulation results (see Figure 4B).

These results were the first indication that the receptor morphology plays a relatively minor role in the binding kinetics compared to the dimensionality of motion of receptors, i.e., whether receptors diffuse in three-dimensional solution or on the surface of a cell. To further analyze these findings, we decided to establish a detailed quantitative mapping between the macroscopic and microscopic binding rates.

### 3.3 Quantitative Mapping of the Macroscopic and Microscopic Binding Rates Reveals Impact of Dimensionality of Motion

**Figure 5:**
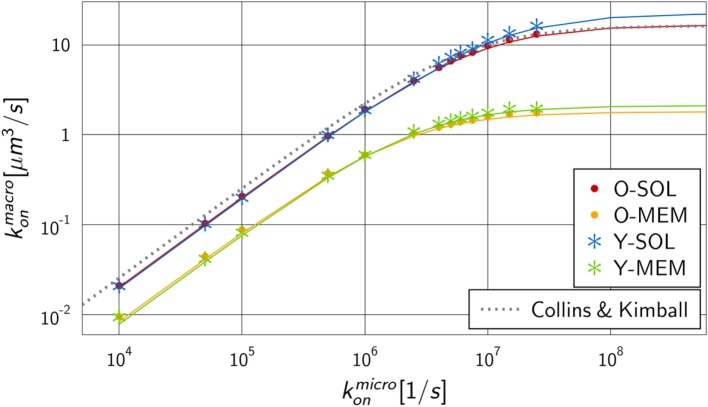
Mapping of microscopic and macroscopic binding rates for different ABM variants. Simulation of all four ABM variants for varying $k_{on}^{micro}$ and the fitted reaction rate $k_{on}^{\mathrm{macro}}$ of the ODE models. Solid lines represent Hill functions with parameters fitted to the data points $k_{on}^{\mathrm{macro}}\left(k_{on}^{micro}\right)$. Results for ABM variants are similar for the same dimensionality of motion for receptors, i.e., either in solution (O-SOL, Y-SOL) or membrane anchored (O-MEM, Y-MEM), but are distinct for ABM variants with soluble and membrane-anchored receptors. The dotted line represents the binding rate as determined by Collins and Kimball (see equation (14)) that is, as expected, comparable to the simulation result for ABM variant O-SOL.

We performed numerical simulations to quantify the difference in monovalent RL binding as a function of receptor properties. All four ABM variants were applied using the fixed dissociation rate $k_{off}^{micro}\;=\;k_{off}^{macro}=0.1\;\;{\text{s}}^{-1}$ and varying the microscopic binding rate in the range $10^{4}\;{\text{s}}^{-1}\leq k_{on}^{micro}\leq 2.5\times 10^{7}\;{\text{s}}^{-1}$. The corresponding macroscopic binding rate $k_{on}^{macro}$ was determined for each numerical experiment from the best fit of the analytical solution of the ODE model to the simulation result of the ABM. The resulting function $k_{on}^{macro}\left(k_{on}^{micro}\right)$ is shown in Figure 5 for each ABM variant. The steady state concentrations of complexes and receptors obtained by fitting the ODE kinetics to the dynamics of the four various ABM variants are summarized in Tables S3–S6 in Supplementary Material.

As expected from our previous considerations, the quantitative difference between morphologies of monovalent receptors is negligible compared to the dimensionality of motion, i.e., whether receptors were diffusing in solution or within the membrane on the surface of a cell. Moreover, the numerical results $k_{on}^{macro}\left(k_{on}^{micro}\right)$ in Figure 5 resemble Hill functions,

(13) $$k_{on}^{macro}\;\left(k_{on}^{micro}\right)=\frac{a\;k_{on}^{micro}}{b+k_{on}^{micro}},$$

with parameters *a* and *b* that are specific for given receptor properties. Here, *a* denotes the upper limit for the macroscopic binding rate, $k_{on}^{macro}\left(k_{on}^{micro}\gg b\right)\to a$, and *b* is a constant that determines the slope of the Hill function, $k_{on}^{macro}\left(k_{on}^{micro}\ll b\right)\to \left(a∕b\right)k_{on}^{micro}$, while at intermediate value $k_{on}^{micro}=b$ the Hill function attains half of its maximal value: $k_{on}^{macro}\left(k_{on}^{micro}=b\right)=a∕2$. The two parameters can be determined from a fit to the numerical simulations and the resulting curves are shown in Figure 5 as solid lines. The corresponding values are summarized in Table S7 in Supplementary Material for the four ABM variants.

The observed functional dependence of $k_{on}^{macro}$ on $k_{on}^{micro}$ is in agreement with theoretical considerations by Collins and Kimball on binding reactions of diffusing receptors and ligands in three spatial dimensions (31–33). They arrived at the expression

(14) $$k_{on}^{macro}\left(\kappa \right)=\frac{k_{s}\;\kappa }{k_{s}+\kappa },$$

where $k_{s}=4\pi \left(r_{L}+r_{R}\right)\left(D_{L}+D_{R}\right)$ denotes the diffusion-controlled reaction rate that was previously introduced by Von Smoluchowski (34) and that depends on the radii of receptor (*r*_R_) and ligand (*r*_L_) as well as on the diffusion coefficients of receptor (*D*_R_) and ligand (*D_L_*). This rate refers to the frequency at which diffusing receptors and ligands come into contact, i.e., have the distance *r_R_* + *r_L_*. Furthermore, κ denotes the intrinsic reaction rate, $\kappa =V_{r}\;k_{on}^{micro}$, which is directly related to the microscopic binding rate $k_{on}^{micro}$ and the reaction volume $V_{r}=\left(4∕3\right)\pi {\left(r_{L}+r_{R}\right)}^{3}$ (35, 36). Combining equations (13) and (14) yields the following relationships:

(15) $$a=k_{s},$$

(16) $$b=\frac{k_{s}}{V_{r}}.$$

It should be stressed that this correspondence can strictly speaking only be applied to monovalent receptors with spherical morphology and to RL binding in three-dimensional solution with receptor and ligand being allowed to penetrate each other. In other words, equations (15) and (16) could only be expected to hold for the ABM variant O-SOL, however, even this scenario is different from the theoretical considerations in that molecules are not allowed to penetrate each other in our ABM. In the ABM, we generally do not allow for molecular penetration in RL interactions, which reduces their possible overlap to a point contact. The implementation of push-back collisions between molecules effectively reduces the reaction volume *V_r_*, i.e., we set *V_r_* → *f_r_V*_r_ with scaling factor *f_r_* ≤ 1. This parameter will only affect the slope of the Hill function, while it was observed in Figure 5 that the upper limit of the macroscopic binding rate, *k_s_*, does as well depend on the receptor properties. To account for these observations, we set *k_s_* → *f_s_k_s_* with scaling factor *f_s_*. It then follows that *f_r_* and *f_s_* can be computed from the equations

(17) $$f_{s}=\frac{a}{k_{s}},$$

(18) $$f_{r}=\frac{f_{s}\;k_{s}}{b\;V_{r}}$$

in terms of the two fitting parameters *a* and *b* (see Table S7 in Supplementary Material). The resulting scaling factors are summarized in Table S8 in Supplementary Material.

As could be expected, for the ABM variant O-SOL we found the scaling factor $f_{s}^{\;O-SOL}=1.02$ to be close to 1, implying that the upper limit for the macroscopic binding rate as predicted by Collins and Kimball was quantitatively recovered (31–33). Regarding the increase of $k_{on}^{macro}$ as a function of $k_{on}^{micro}$, we found the difference in the underlying assumptions on RL interactions to be reflected by a decrease in the reaction volume *V_r_* with scaling factor $f_{r}^{\;O-SOL}=0.79$.

**Figure 6:**
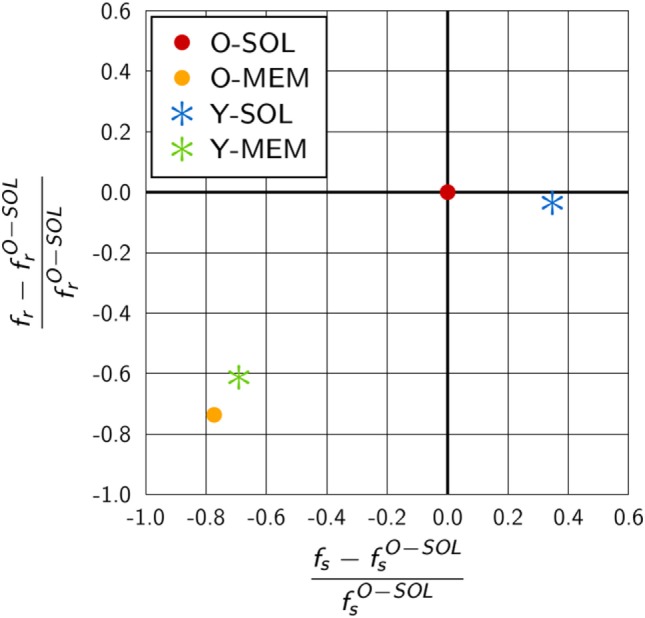
Quantitative difference in the scaling factors of ABM variants relative to O-SOL. The scaling factors *f_s_* and *f_r_* are calculated from equations (17) and (18) for parameters specific to the considered ABM variant. ABM variant O-SOL resembles the conditions of the theoretical considerations by Collins and Kimball (31–33) most of all. Scaling factors of ABM variants with membrane-anchored receptors that are either spherically shaped (O-MEM) or Y-shaped (Y-MEM) exhibit similar but clear differences to ABM variant O-SOL, whereas ABM variant Y-SOL is most similar to O-SOL.

We compared the scaling factors for the other ABM variants and present the results relative to ABM variant O-SOL in Figure 6. The scaling factor $f_{r}^{\;Y-SOL}$ of ABM variant Y-SOL was found to be similar to $f_{r}^{\;O-SOL}$ with a relative decrease of only 4%, whereas this scaling factor for the ABM variants with membrane-anchored receptors, i.e., $f_{r}^{\;O-MEM}$ and $f_{r}^{\;Y-MEM}$, was decreased by 74 and 61%, respectively. Furthermore, as shown in Figure 6, the scaling factors $f_{s}^{\;O-MEM}$ and $f_{s}^{\;Y-MEM}$ for membrane-anchored receptors were found to be decreased from $f_{s}^{\;O-SOL}$ by 77 and 69%, respectively, indicating a significant change in the upper limit of the macroscopic binding rate. On the other hand, this scaling factor was always somewhat higher for membrane-anchored receptors, i.e., ABM variants O-MEM and Y-MEM, compared to their respective counterparts with soluble receptors.

We checked the dependency of the mapping between macroscopic and microscopic binding rates (see Figure 5) as well as the scaling factors *f_s_* and *f_r_* (see Figure 6) on the down-scaling factor *s* of the simulated ABM variants. It was generally observed that simulations for soluble receptors were not affected by the system down-scaling, whereas in simulations for membrane-anchored receptors increasing the down-scaling factor *s* resulted into lower values for $k_{on}^{macro}$ as a function of $k_{on}^{micro}$. This implies that the difference between ABM variants with soluble and membrane-anchored receptors as observed in Figure 5 as well as the distances between the respective scaling factors in Figure 6 represents a lower limit.

Since the diffusion coefficients of receptors in the soluble (*D_R_* = 90 µm^2^ s^−1^) and membrane-anchored (*D_R_* = 0.05 µm^2^ s^−1^) variant differed by orders of magnitude, we checked whether differences in the upper limit of the macroscopic binding rate were indeed merely a consequence of the dimensionality of motion rather than of the magnitude of the diffusion coefficient itself. This was done by running simulations with interchanged diffusion coefficients, i.e., ABM variant O-SOL with *D_R_* = 0.05 µm^2^ s^−1^ and ABM variant O-MEM with *D_R_* = 90 µm^2^ s^−1^. However, even this dramatic modification of diffusion coefficients did not eliminate the significant difference in the dependence of $k_{on}^{macro}$ on $k_{on}^{micro}$ between the ABM variants (see Figures S2 and S3 in Supplementary Material).

Taken together, our quantitative analysis of monovalent RL binding kinetics revealed the impact of receptor properties on the macroscopic binding rate and by that on the association constant of the RL binding. It was shown that the diffusion coefficients of receptors and their morphology have minor effects, whereas the strongest impact was due to the dimensionality of motion. Compared to soluble receptors in three dimensions, RL binding kinetics of membrane-anchored receptors on a cellular surface were retarded and could not achieve comparably high association constants. In what follows, we consider the impact of the binding valency by taking into account that the Y-shaped receptors can bind a ligand at each receptor arm.

### 3.4 Binding Valency Reduces Differences in the Binding Kinetics of BCR and Antibodies

**Figure 7:**
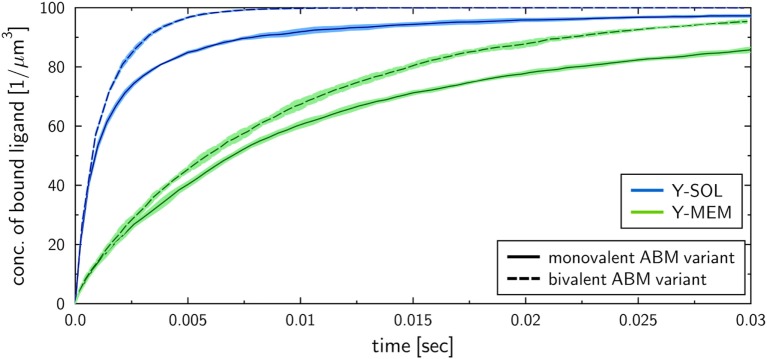
Kinetics of bound ligands for Y-MEM and Y-SOL ABM variants with either monovalent or bivalent receptors. Time-dependent concentration of bound ligands for ABM variants Y-MEM and Y-SOL for models with either monovalent receptors or bivalent receptors. All models were simulated with dissociation rate $k_{off}^{micro}=0.1\;\;{\text{s}}^{-1}$ and binding rate $k_{on}^{micro}=10^{7}\;{\text{s}}^{-1}$. Dark and pale lines in different colors represent, respectively, mean values and standard deviations of five simulation runs per ABM variant.

**Figure 8:**
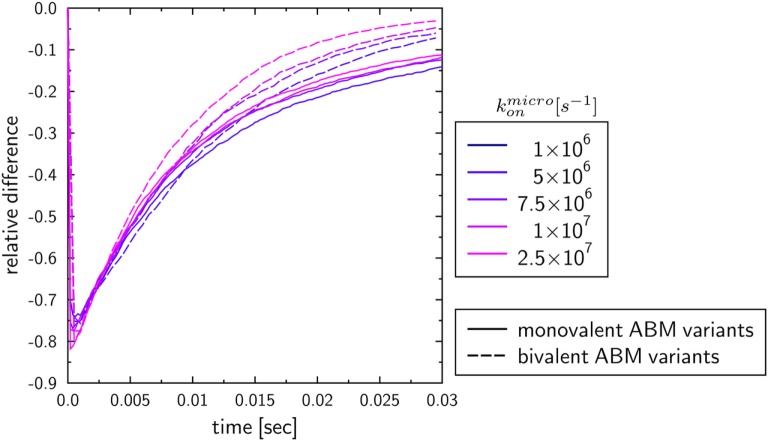
Relative differences between ABM variants Y-MEM and Y-SOL with either monovalent or bivalent receptors. Temporal evolution of the relative differences of bound ligands between ABM variants Y-MEM and Y-SOL for models with either monovalent receptors (monovalent ABM variant) or bivalent receptors (bivalent ABM variant). The colors refer to ABM variants with varying binding rates $k_{on}^{micro}$.

To investigate the influence of the receptor binding valency on the binding kinetics for monovalent receptors (see Figure 5), we modified ABM variants Y-MEM and Y-SOL as to allow for bivalent binding of the Y-shaped receptors, i.e., a ligand can bind at each of the two receptor arms. Thus, in these ABM variants the term complex refers to receptors that are bound to either one or two ligands. The simulations were performed with varied binding rate $k_{on}^{micro}$ between 5 × 10^6^ and 2.5 × 10^7^ s^−1^. The temporal course of the binding kinetics for simulations of the bivalent and monovalent ABM variants is shown in Figure 7. The simulations of $k_{on}^{micro}=1\times 10^{7}\;{\text{s}}^{-1}$ exhibit the typical relations between the binding kinetics of the ABM variants. As could be expected, both ABM variants with bivalent receptors showed a faster binding kinetics and also reached higher association constants than their monovalent counterparts. In Figure 8, we show the relative difference in receptor-bound ligands for ABM variant Y-MEM relative to ABM variant Y-SOL and for different values of $k_{on}^{micro}$. This difference is significantly smaller (down to 72%) for bivalent receptors compared to monovalent receptors, and in the limit of long times this difference vanishes only for bivalent but not for monovalent receptors. These results indicate that the binding valency makes a clear difference for RL binding: In the case of monovalent receptors, the dimensionality of motion induces a significant difference in the binding kinetics, whereas this difference is largely compensated by the bivalency of receptors. Thus, it turns out that membrane-anchored BCR and soluble antibodies do reach comparable association constants for bivalent receptors.

In order to investigate whether these observations are caused by the effectively twofold number of binding sites for the bivalent receptors, we performed simulations with ABM variants that have twice as much monovalent receptors than the so far applied physiological number of receptors $\left(N_{p}^{R}\right)$. The binding kinetics of ABM variants with $N^{R}=2\times N_{p}^{R}$ monovalent receptors turned out to be even faster as the binding kinetics of bivalent ABM variants with $N^{R}=N_{p}^{R}$ (see Figure S4 in Supplementary Material). Additionally, the relative differences between binding kinetics of ABM variants with soluble and membrane-bound receptors vanishes with increasing time, and this occurs slightly faster as for ABM variants with bivalent receptors (see Figure S5 in Supplementary Material). These results indicate that comparable association constants of membrane anchored and soluble receptors can be observed for systems with higher amounts of binding sites at receptors.

## 4 Discussion

The focus of this study on receptor–ligand (RL) binding was twofold. Firstly, we established a quantitative mapping between macroscopic binding rates of an ordinary differential equation (ODE) model and their microscopic equivalents as obtained from simulating the spatiotemporal binding kinetics by agent-based models (ABM). Secondly, we investigated the impact of various properties of B cell-derived receptors—such as their dimensionality of motion, morphology and binding valency—on the RL binding kinetics.

Regarding the quantitative mapping of binding rates, we recovered for fixed dissociation rates $k_{off}^{micro}=k_{off}^{macro}=0.1\;\;{\text{s}}^{-1}$ the non-linear relationship between the binding rates $k_{on}^{macro}$ and $k_{on}^{micro}$. This resembles a Hill-type function (see Figure 5), which is in line with theoretical predictions by Collins and Kimball (31–33). Scanning $k_{on}^{micro}$ over more than four orders of magnitude, we obtained upper limiting values for $k_{on}^{macro}$ in the range 10^0^–10^1^ µm^3^ s^−1^, which corresponds to 10^8^–10^9^ M^−1^ s^−1^ using Avogadro’s number. For $k_{off}^{macro}=0.1\;{\text{s}}^{-1}$, the resulting association constant is *K_a_* = 10^10^ M^−1^. This is in agreement with experimentally measured values for BCR-antigen binding, where typical values up to *K_a_* = 10^10^ M^−1^ are reached (37, 38), which is a strong indication for our ABM variants to be realistic and quantitative to-scale representations of RL binding.

The ABM variants were implemented in three-dimensional representations of continuous space and RL binding was simulated by the random selection method (5). We implemented different ABM variants where binding of spherical ligands occurs either with soluble receptors or with membrane-anchored receptors. The receptors are either spherically shaped or Y-shaped and can be mono- or bivalent. We simulated RL binding in identical environments to allow for quantitative comparisons of the different scenarios. In particular, we considered the Y-shaped and bivalent antibodies in solution and the B cell receptors (BCR) as their membrane-anchored counterparts on a spherical cell to be an appropriate example. In previous work on BCR binding, ABM implementations typically involved simplifications with regard to the spatial representation, i.e., using a planar cell surface and imposing a spatial grid for molecule diffusion (39, 40) and have been applied to simulate the immunological synapse involving B cells (41–45) or T cells (46, 47). Besides this work on immune cell receptor–ligand interaction, there exist software packages for the simulation of various type, such as Smoldyn (48) and MCell (49, 50). Even though these simulators represent molecular diffusion in lattice-free continuous space, they lack features that are essential in the present study. For example, Smoldyn represents molecules in a point-like fashion (48, 51–53), while MCell does only allow to determine an upper limit of the simulation time step Δ*t* (54) implying that simulations with different model systems may differ in the time step Δ*t*. Therefore, we did not consider these simulators suitable for the investigation of morphological aspects of receptors and for comparing models at the microscopic and macroscopic scale. Moreover, the RL binding of soluble and membrane-anchored receptors was previously also investigated by non-spatial ODE models (55, 56). These two-step ODE models comprise the process of encounter formation by molecule diffusion and the reaction process itself, so that molecular parameters, like diffusion constant and size, could also be incorporated. However, several simplifications were made, such as the derivation of the binding rate of membrane-bound receptors from cell–ligand interaction rate, which turned out to be not applicable in general (55, 56).

To study the impact of various receptor properties on RL binding kinetics, we compared scenarios that differ in the dimensionality of motion, morphology and binding valency of receptors. These receptor properties were investigated since they are characteristic for B cell-derived receptors that play a key role in the adaptive immune response. Interestingly, the RL binding kinetics for monovalent Y-shaped receptors was observed to be quantitatively comparable to that of spherical receptors (see Figure 5), i.e., the difference in the morphology of monovalent receptors did not reveal a substantial impact. In contrast, the dimensionality of motion for BCR compared to soluble antibodies did reveal a clear difference in the binding kinetics, i.e., the association constants were found to be significantly lower for membrane-anchored receptors compared to soluble receptors (see Figure 5). Furthermore, our results show that the diffusion constant of receptors, which is much smaller for membrane-anchored molecules as for soluble molecules, does not strongly influence the observed differences in the binding kinetics. This suggest that the difference in the association constants for soluble and membrane-anchored monovalent receptors originate from the difference in the dimensionality of motion. However, this difference was largely compensated by taking into account that BCR and soluble antibodies are bivalent (see Figure 8), i.e., the relative difference in the binding kinetics of membrane-anchored and soluble receptors vanished only in the case of bivalent receptors. It is generally known that the bivalency of BCR supports cross-linking in the binding to multivalent ligands. However, the current findings suggest that the bivalency of BCR does also compensate the difference in the association constant that exist for monovalent receptors between the soluble and membrane-anchored variants.

In the future, the extensibility of the current simulation framework can be exploited to study more complex scenarios. For example, antigens may be represented by multivalent ligands that do not only allow for cross-linking of BCR but also binding to coreceptors required for B cell activation. This enables to study the important process of BCR clustering on the cell surface (57–59) that has also been the subject of theoretical investigations (39, 40, 60, 61). We envisage that such studies will strongly benefit from an image-based systems biology approach, for example, as applied by Mech et al. (62) and conceptionally reviewed by Medyukhina et al. (63). Recently, we took the first steps toward an image-based investigation of B cell activation that requires the concerted action of various receptors and ligands (64). Based on these data, our ABM can be extended by various agent types with specific properties to predict prerequisites for experimentally observed molecular patterns. Moreover, the ABM variants could be modified to represent various receptor properties of different antibody isotypes and/or subclasses, which would allow investigating the impact of specific receptor properties on the RL binding kinetics. Based on this modification, the impact of naturally occurring antibody complexes, such as IgA dimers and IgM pentamers, could be investigated. Furthermore, extending the ABM to represent arbitrarily shaped cells that are brought in close contact, it can be used to simulate the molecular patterns during synapse formation involving B cells, T cells as well as phagocytes (65–68). This would enable to investigate the impact of the dimensionality of motion of ligands that is reported to be an important parameter for regulating B cell activation and signaling (69).

## Author Contributions

TL and MF conceived and designed the study, evaluated and analyzed the results, and wrote the manuscript and critically revised it. MF contributed materials and computational resources. TL processed the data, implemented, and applied the computational algorithm.

## Conflict of Interest Statement

The authors declare that the research was conducted in the absence of any commercial or financial relationships that could be construed as a potential conflict of interest.

## Supplementary Material

Video S1 Simulation of down-scaled scaled O-SOL ABM variant. ABM simulation with monovalent receptors (blue objects) that are spherically shaped and move in solution by performing three-dimensional diffusion. Upon contact between receptors and ligands (red objects) these may bind and form RL complexes (green objects) depending on the binding rate $k_{on}^{micro}$ = 2.5 × 10^7^ s^−1^. The system is down-scaled with factor s = 0.01 (see Supplementary Material) and values of model parameters are provided in Tables S1 and S2 in Supplementary Material. The video is composed of 15 frames s^−1^ and the simulation time between two consecutive frames is 6.8 × 10^−8^ s. A high-resolution video is available for download from https://asbdata.hki-jena.de/LehnertFigge2017_FrontImmun/.

Video S2 Simulation of down-scaled O-MEM ABM variant. ABM simulation with monovalent receptors (blue objects) that are spherically shaped and move in the cell membrane by performing two-dimensional diffusion. Upon contact between receptors and ligands (red objects) these may bind and form RL-complexes (green objects) depending on the binding rate $k_{on}^{micro}$ = 2.5 × 10^7^ s^−1^. The system is down-scaled with factor s = 0.01 (see Supplementary Material) and values of model parameters are provided in Tables S1 and S2 in Supplementary Material. The video is composed of 15 frames s^−1^ and the simulation time between two consecutive frames is 6.8 × 10^−8^ s. A high-resolution video is available for download from https://asbdata.hki-jena.de/LehnertFigge2017_FrontImmun/.

Video S3 Simulation of down-scaled Y-SOL ABM variant. ABM simulation with monovalent receptors (blue objects) that are Y-shaped and move in solution by performing three-dimensional diffusion. Upon contact between receptors and ligands (red objects) these may bind and form RL-complexes (green objects) depending on the binding rate $k_{on}^{micro}$ = 2.5 × 10^7^ s^−1^. The system is down-scaled with factor s = 0.01 (see Supplementary Material) and values of model parameters are provided in Table S1 in Supplementary Material and Supplementary Material. The video is composed of 15 frames s^−1^ and the simulation time between two consecutive frames is 6.8 × 10^−8^ s. A high-resolution video is available for download from https://asbdata.hki-jena.de/LehnertFigge2017_FrontImmun/.

Video S4 Simulation of down-scaled Y-MEM ABM variant. ABM simulation with monovalent receptors (blue objects) that are Y-shaped and move in the cell membrane by performing two-dimensional diffusion. Upon contact between receptors and ligands (red objects) these may bind and form RL-complexes (green objects) depending on the binding rate $k_{on}^{micro}$ = 2.5 × 10^7^ s^−1^. The system is down-scaled with factor s = 0.01 (see Supplementary Material) and values of model parameters are provided in Tables S1 and S2 in Supplementary Material. The video is composed of 15 frames s^−1^ and the simulation time between two consecutive frames is 6.8 × 10^−8 ^s. A high-resolution video is available for download from https://asbdata.hki-jena.de/LehnertFigge2017_FrontImmun/.

Video S5 Simulation of Y-MEM ABM variant. ABM simulation with monovalent receptors (blue objects) that are Y-shaped and move in the cell membrane by performing two-dimensional diffusion. Upon contact between receptors and ligands (red objects) these may bind and form RL-complexes (green objects) depending on the binding rate $k_{on}^{micro}$ = 2.5 × 10^7^ s^−1^. The system is down-scaled with factor s = 0.01 (see Supplementary Material) and values of model parameters are provided in Table S1 in Supplementary Material. The video is composed of 15 frames s^−1^ and the simulation time between two consecutive frames is 6.8 × 10^−8^ s. A high-resolution video is available for download from https://asbdata.hki-jena.de/LehnertFigge2017_FrontImmun/.
