# Supplementary material for: Dimensionality of Motion and Binding Valency Govern Receptor–Ligand Kinetics As Revealed by Agent-Based Modeling
Source: Front Immunol. 2017 Nov 30;8:1692. doi: 10.3389/fimmu.2017.01692 (PMC5714874; doi:10.3389/fimmu.2017.01692)
Supplement: Supplementary file 6 [file Presentation_1.PDF]

Supplementary Material: Dimensionality of motion and binding valency govern receptor-ligand kinetics as revealed by agent-based modeling

Teresa Lehnert and Marc Thilo Figge*
*Correspondence:
Author Name: Marc Thilo Figge
thilo.figge@leibniz-hki.de

1 SUPPLEMENTARY DATA

1.1 Videos

Video S1. Simulation of down-scaled scaled O-SOL ABM variant. ABM simulation with monovalent receptors (blue objects) that are spherically-shaped and move in solution by performing three-dimensional diffusion. Upon contact between receptors and ligands (red objects) these may bind and form RL-complexes (green objects) depending on the binding rate $k_{on}^{micro} = 2.5 \times 10^7\ s^{-1}$. The system is down-scaled with factor $s = 0.01$ (see Supplementary Information) and values of model parameters are provided in Table S1 and Table S2 in Supplementary Material. The video is composed of 15 frames per second and the simulation time between two consecutive frames is $6.8 \times 10^{-8}\ s$.
A high-resolution video is available for download from `https://asbdata.hki-jena.de/LehnertFigge2017_FrontImmun/`.

Video S2. Simulation of down-scaled O-MEM ABM variant. ABM simulation with monovalent receptors (blue objects) that are spherically-shaped and move in the cell-membrane by performing two-dimensional diffusion. Upon contact between receptors and ligands (red objects) these may bind and form RL-complexes (green objects) depending on the binding rate $k_{on}^{micro} = 2.5 \times 10^7\ s^{-1}$. The system is down-scaled with factor $s = 0.01$ (see Supplementary Information) and values of model parameters are provided in Table S1 and Table S2 in Supplementary Material. The video is composed of 15 frames per second and the simulation time between two consecutive frames is $6.8 \times 10^{-8}\ s$.
A high-resolution video is available for download from `https://asbdata.hki-jena.de/LehnertFigge2017_FrontImmun/`.

Video S3. Simulation of down-scaled Y-SOL ABM variant. ABM simulation with monovalent receptors (blue objects) that are Y-shaped and move in solution by performing three-dimensional diffusion. Upon contact between receptors and ligands (red objects) these may bind and form RL-complexes (green objects) depending on the binding rate $k_{on}^{micro} = 2.5 \times 10^7\ s^{-1}$. The system is down-scaled with factor $s = 0.01$ (see Supplementary Information) and values of model parameters are provided in Table S1 and Supplementary Information. The video is composed of 15 frames per second and the simulation time between two consecutive frames is $6.8 \times 10^{-8}\ s$.
A high-resolution video is available for download from `https://asbdata.hki-jena.de/LehnertFigge2017_FrontImmun/`.

Video S4. Simulation of down-scaled Y-MEM ABM variant. ABM simulation with monovalent receptors (blue objects) that are Y-shaped and move in the cell-membrane by performing two-dimensional diffusion. Upon contact between receptors and ligands (red objects) these may bind and form RL-complexes (green objects) depending on the binding rate $k_{on}^{micro} = 2.5 \times 10^7\, s^{-1}$. The system is down-scaled with factor $s = 0.01$ (see Supplementary Information) and values of model parameters are provided in Table S1 and Table S2 in Supplementary Material. The video is composed of 15 frames per second and the simulation time between two consecutive frames is $6.8 \times 10^{-8}\, s$.
A high-resolution video is available for download from `https://asbdata.hki-jena.de/LehnertFigge2017_FrontImmun/`.

Video S5. Simulation of Y-MEM ABM variant. ABM simulation with monovalent receptors (blue objects) that are Y-shaped and move in the cell-membrane by performing two-dimensional diffusion. Upon contact between receptors and ligands (red objects) these may bind and form RL-complexes (green objects) depending on the binding rate $k_{on}^{micro} = 2.5 \times 10^7\, s^{-1}$. The system is down-scaled with factor $s = 0.01$ (see Supplementary Information) and values of model parameters are provided in Table S1 in Supplementary Material. The video is composed of 15 frames per second and the simulation time between two consecutive frames is $6.8 \times 10^{-8}\, s$.
A high-resolution video is available for download from `https://asbdata.hki-jena.de/LehnertFigge2017_FrontImmun/`.

2 SUPPLEMENTARY INFORMATION

Molecular diffusion. All molecules in the ABM perform diffusive motion that is characterized by the diffusion coefficient D. Depending on the dimensionality of motion, *e.g.* in the case of membrane-anchored receptors on the surface of the cell membrane or in the three spatial dimensions of solution, the value of diffusion coefficients do vary. For example, for membrane-anchored molecules the typical range is reported to be $D = 0.01 - 0.1\, \mu m^2 s^{-1}$ of membrane-anchored molecules (1, 2, 3) and in the simulations we set the diffusion coefficient of these receptors to the typical value $D = 0.05\, \mu m^2 s^{-1}$. Diffusion coefficients in three-dimensional solution can be estimated using the Stokes-Einstein equation (4):

$$D = \frac{k_B T}{6\pi\eta r}\,. \tag{S1}$$

Here, k_B denotes the Boltzmann constant, η is the viscosity of the solution and T its temperature, while r refers to the radius of molecules under consideration. The corresponding diffusion coefficients of membrane-anchored receptors are listed in Table S1 in Supplementary Material.

Relation between microscopic and macroscopic dissociation rates. The relation between the rate for microscopic dissociation of complexes and its macroscopic counterpart is described differently in several studies. On the one hand, some studies assume that macroscopic dissociation of a molecular complex is a spontaneous process of a single molecule. This process is measured by the inverse mean time needed for the complex to decay into its compartments, so that k_{off}^{micro} equals the experimentally measured k_{off}^{macro} (5, 6, 7, 8, 9). On the other hand, in the theory of Eigen (10), applied by other studies (11, 12, 13, 14, 15, 16), it is assumed that macroscopic dissociation of molecules is composed of two consecutive processes. First, the complex breaks into its molecular components, but are still in contact and form an encounter complex. Second, the molecules of the encounter complex have to diffuse away

from each other to prevent immediate rebinding. However, it should be noted that the resulting mapping between the macroscopic dissociation rate and the rates of the consecutive processes is based on the assumption that the concentration of encounter complexes is in steady state.

For our model system we first checked whether the prerequisite is met that encounter complexes in the simulations are indeed in steady state. Since we vary the value of k_{on}^{micro} in this study, we checked the time-dependent number of encounter complexes in simulations with $k_{on}^{micro} = 10^5\,s^{-1}$, $10^6\,s^{-1}$ and $2.5 \times 10^7 s^{-1}$ keeping the value of the dissociation rate constant at $k_{off}^{micro} = 0.1s^{-1}$. In Figure S4 in Supplementary Material it can be seen that the initial increase in the number of encounter complexes is followed by a decrease whose slope depends on the microscopic binding rate. These results clearly show that the number of encounter complexes is not constant during the whole simulation time of $0.1s$. This indicates that the steady-state assumption, which is required in the theory of Eigen (10), is not applicable for the parameters of antibody-antigen binding used in our model.

Furthermore, we investigated the impact of rebinding in our model simulations. As a prerequisite for rebinding, the number of dissociation events was computed and is depicted in Figure S5 in Supplementary Material. Here, it can be seen that the accumulated number of dissociation events increases between simulations with increasing microscopic binding rate k_{on}^{micro}, which follows from the higher number of RL-complexes that can dissociate. After simulation time of $0.1s$, even in simulations with relatively high binding rate $k_{on}^{micro} = 2.5 \times 10^7\,s^{-1}$, only 10 ± 2.6 dissociation events were detected out of the total amount of 906 ± 1.8 formed RL-complexes. This shows that the resulting dissociation rate of $k_{off}^{micro} = 0.11 \pm 0.03\,s^{-1}$ reproduces the input value ($k_{off}^{micro} = 0.1s^{-1}$) and implies that dissociation is a very rare process in our model simulations, where only 1% of around 1000 RL-complexes dissociate at maximum. Consequently, rebinding events do not substantially affect the binding dynamics of our model system and we therefore set $k_{off}^{micro} = k_{off}^{macro}$ in this study.

Down-scaling of the model system. To make simulations of the ABM computationally feasible, we decrease the number of molecules keeping the molecular concentration fixed. This implies that the system size, *i.e.* the cell size and the size of the environment, has to be down-scaled appropriately. We down-scale the number of receptor molecules n_R with factor $s < 1$ to obtain the scaled number of receptors as $n_R^s = s\,n_R$, where the superscript "s" denotes the scaled quantity. Using the condition of constant concentrations in the real and the down-scaled systems, it is straightforward to show that the radius r_C of the cell scales as $r_C^s = \sqrt{s}\,r_C$. Similarly, the number of ligands is down-scaled by the factor s: $n_L^s = s\,n_L$. For the radius of the down-scaled spherical environment, r_E^s, this implies

$$r_E^s = \sqrt[3]{s\,(r_E^3 - r_C^3) \, + \, (\sqrt{s} r_C)^3}. \tag{S2}$$

in terms of the original radius r_E of the environment. The values of the scaled parameters are listed in Table S2 in Supplementary Material.

3 SUPPLEMENTARY TABLES AND FIGURES

3.1 Figures

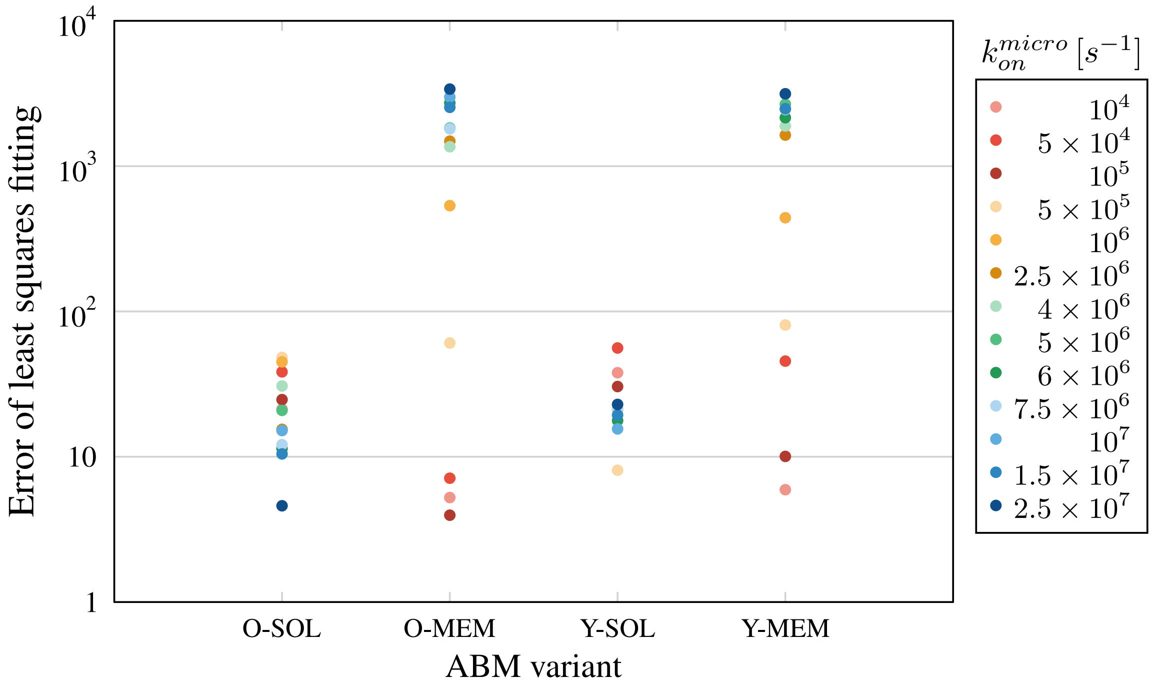

Figure S1. Error of least-squares fitting for simulations with the different ABM variants and its dependence on k_{on}^{micro}.

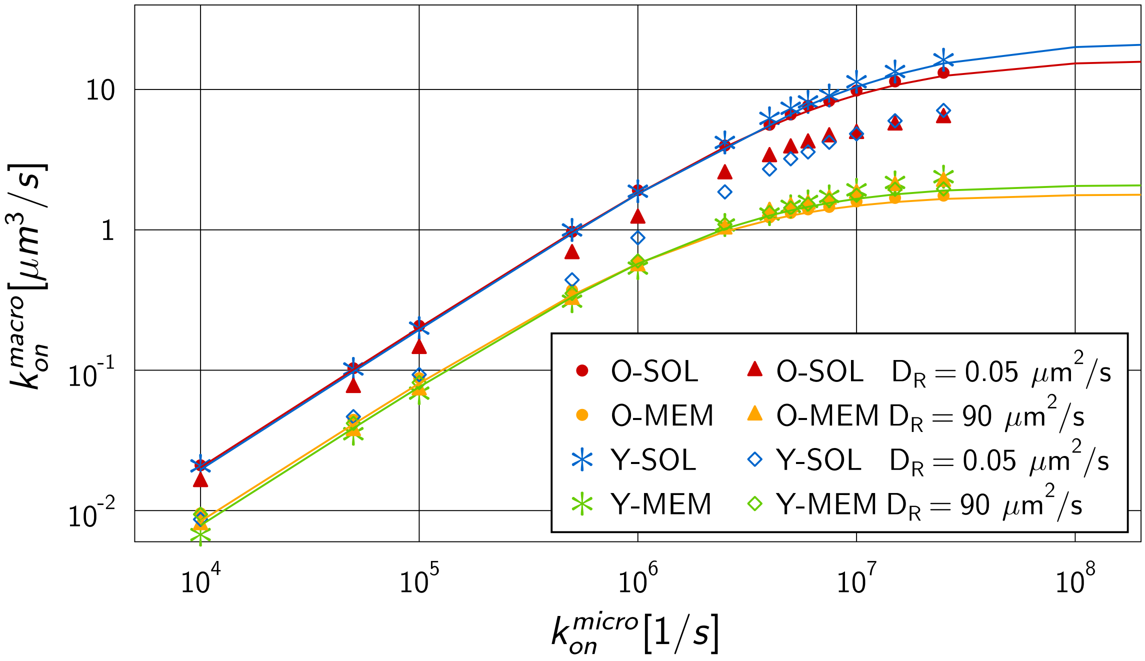

Figure S2. Mapping of microscopic and macroscopic binding rates for different ABM variants with interchanged diffusion coefficients between soluble and membrane-anchored receptors.

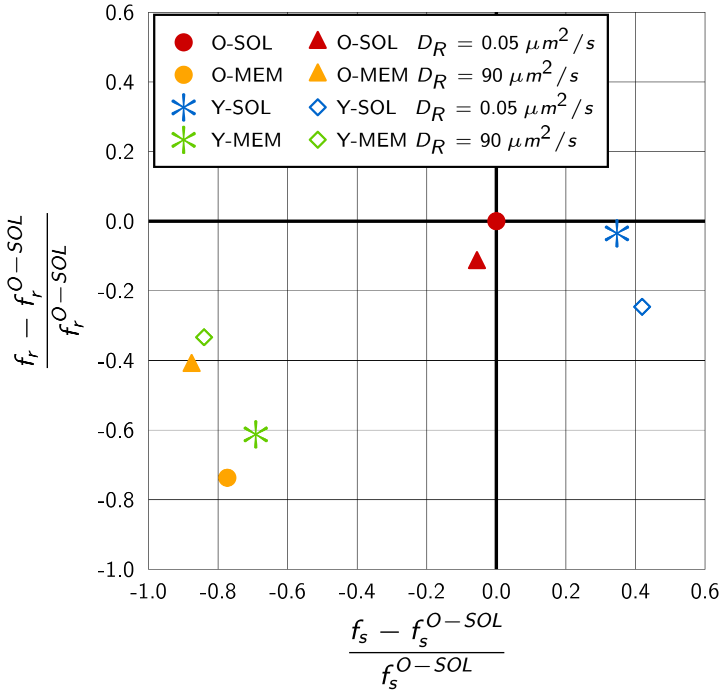

Figure S3. Quantitative difference in the scaling factors of ABM variants relative to O-SOL.

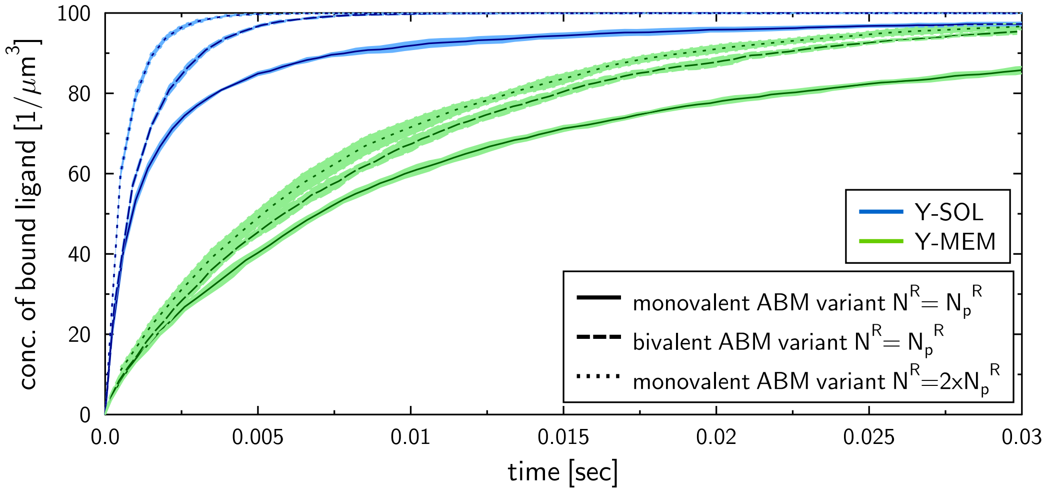

Figure S4. Kinetics of bound ligands for Y-MEM and Y-SOL ABM variants with different numbers of monovalent or bivalent receptors. Time-dependent concentration of bound ligands for ABM variants Y-MEM and Y-SOL for models either with physiological number (N_p^R) monovalent receptors or bivalent receptors, or with twice the physiological number ($2 \times N_p^R$) monovalent receptors. All models were simulated with dissociation rate $k_{off}^{micro} = 0.1\, s^{-1}$ and binding rate $k_{on}^{micro} = 10^7\, s^{-1}$. Dark and pale lines in different colors represent, respectively, mean values and standard deviations of five simulation runs per ABM variant.

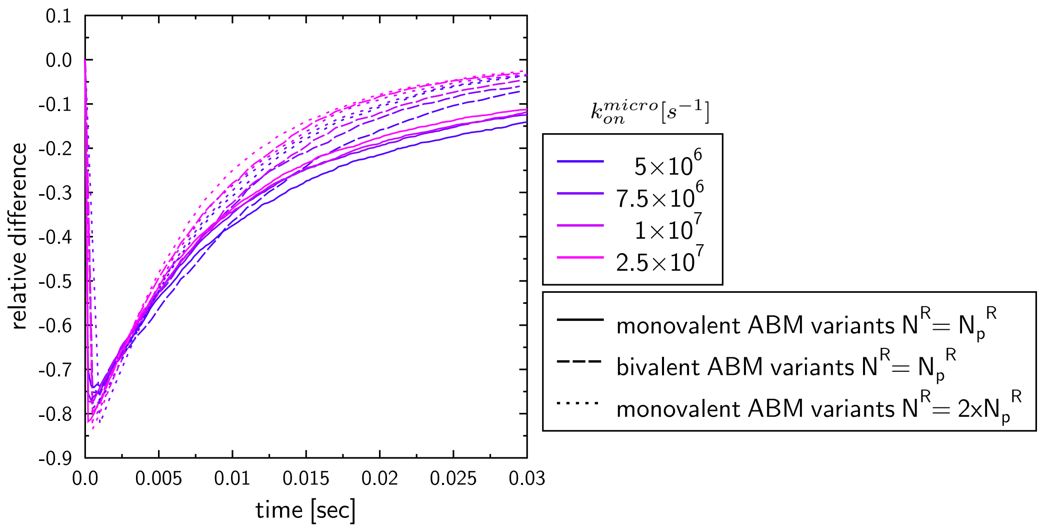

Figure S5. Relative differences between ABM variants Y-MEM and Y-SOL with different numbers of monovalent or bivalent receptors. Temporal evolution of the relative differences of bound ligands between ABM variants Y-MEM and Y-SOL for models either with physiological number (N_p^R) monovalent receptors or bivalent receptors, or with twice the physiological number ($2 \times N_p^R$) monovalent receptors. The colors refer to ABM variants with different binding rates k_{on}^{micro}.

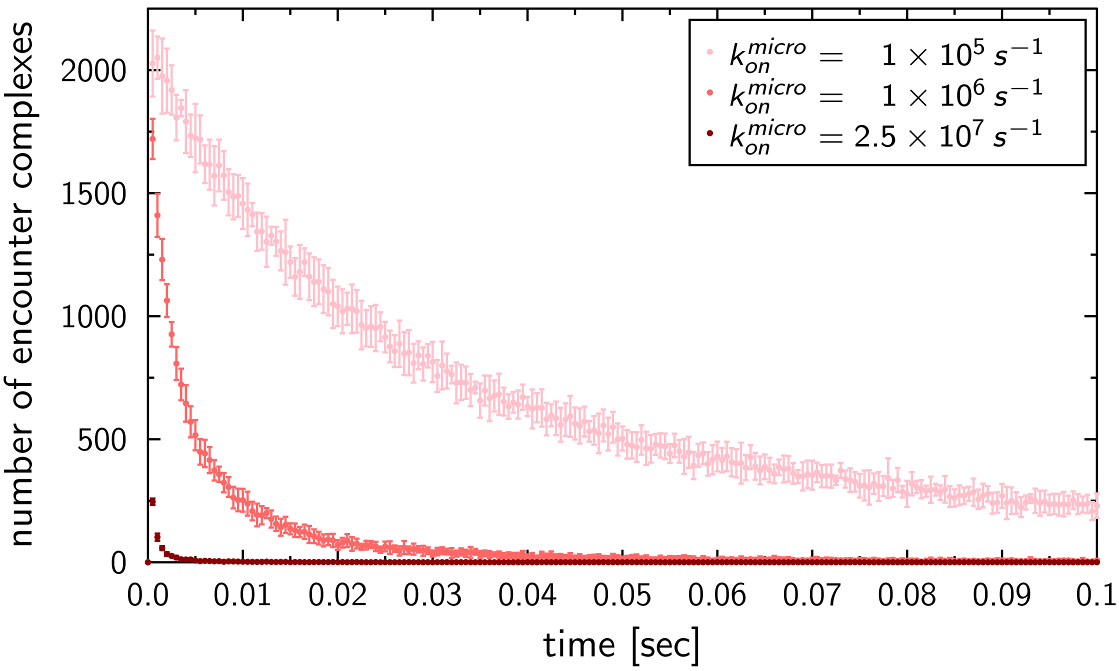

Figure S6. Number of encounter complexes for simulations with O-SOL ABM variant. Mean number of encounter complexes that are formed within recording time step of $\Delta t_{record} = 5 \times 10^{-4} s$. Dots represent the mean values and bars refer to the standard deviation calculated from ten repeated simulations.

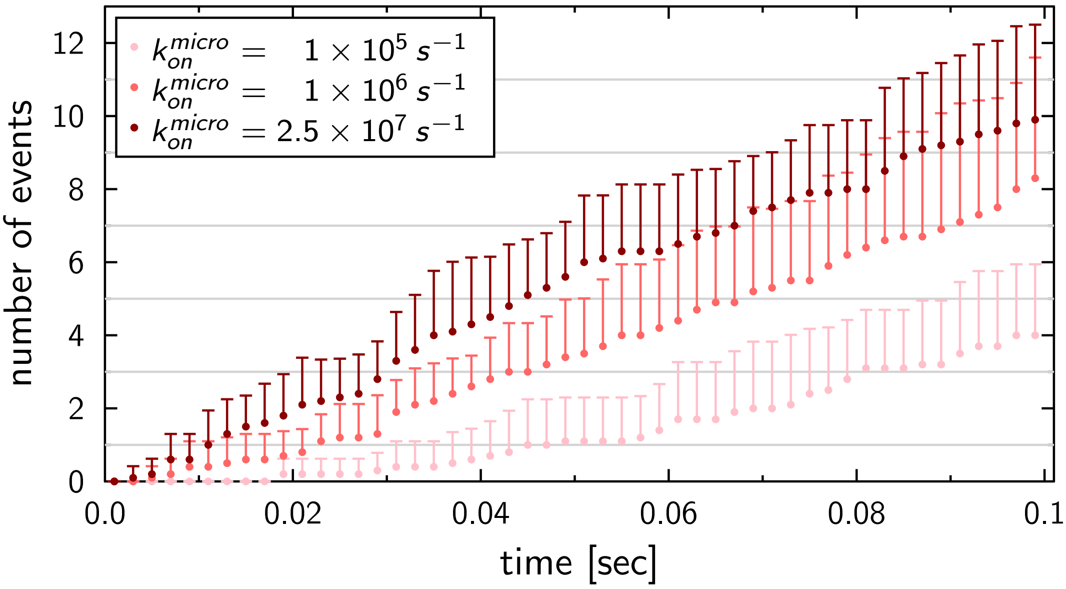

Figure S7. Number of dissociation events for simulations with O-SOL ABM variant. Mean number of accumulated dissociation events resulting from ABM simulations with three different binding rates k_{on}^{micro}. Dots represent the mean values and bars refer to the standard deviation calculated from ten repeated simulations.

3.2 Tables

Table S1. Parameter values of ABM variants. The ABM variants are characterized by different specific parameters that were either extracted from the literature or calculated for the considered model system. Characteristic parameters are the radius (r) of spherical objects, the concentration of molecules (c), the molecule number (n), the radius of the binding sphere (r^{bind}) and the collision sphere (r^{coll}), as well as the diffusion coefficient (D). Y-shaped receptors are further characterized by their width (w_R) and height (h_R) and are composed of a cylindrical stem bearing two cylindrical arms with corresponding radii (r_{stem}, r_{arm}) and heights (h_{stem}, h_{arm}). Additionally, Y-shaped receptors possess collision spheres located on their arms with radius r^{coll}_{arm} as well as at the stem with radius r^{coll}_{stem}.

model component	parameter value	reference	comment
environment	$r = 7.56\,\mu m$		calculated from the condition that ligand and receptor numbers are equal
cell	$r = 6\,\mu m$	(17)	
ligand	$c = 100\,\text{molecules}/\mu m^3$	(18)	
	$n = 90480$		
	$r^{coll} = 5.0\,nm$	(19, 20)	
	$r^{bind} = 5.0\,nm$	(19, 20)	
	$D = 63.1\,\mu m^2/s$	(4)	Stokes-Einstein equation with $\eta = 7 \times 10^{-4} Ns/m^2$ and $T = 37°C$ (see Supplementary Notes)
receptors	$c = 200\,\text{molecules}/\mu m^2$	(5)	
	$n = 90480$		calculated from concentration c at given cell surface
O-SOL receptors	$r^{bind} = 3.5\,nm$		calculated to obtain the same effective binding area as for Y-shaped receptors
	$r^{coll} = 3.5\,nm$		
	$D = 90\,\mu m^2/s$	(4)	Stokes-Einstein equation with $\eta = 7 \times 10^{-4} Ns/m^2$ and $T = 37°C$ (see Supplementary Notes)
Y-SOL receptors	$r^{bind} = 3.5\,nm$	(21, 20, 22, 23)	
	$r^{coll}_{arm} = 3.5\,nm$	(20, 24, 22, 21)	
	$r^{coll}_{stem} = 2.0\,nm$	(20, 24, 22, 21)	
	$r_{stem} = 2.0\,nm$	(20, 24, 22, 21)	
	$r_{arm} = 2.0\,nm$	(20, 24, 22, 21)	
	$h_{stem} = 4.5\,nm$	(20, 24, 22, 21)	
	$h_{arm} = 4.5\,nm$	(20, 24, 22, 21)	
	$w_R = 13.0\,nm$	(24)	
	$h_R = 11.0\,nm$	(24)	
	$D = 90\,\mu m^2/s$	(4)	Stokes-Einstein equation with $\eta = 7 \times 10^{-4} Ns/m^2$ and $T = 37°C$ (see Supplementary Notes)
O-MEM receptors	$r^{bind} = 4.9\,nm$		calculated to obtain the same effective binding area as for Y-shaped receptors
	$r^{coll} = 4.9\,nm$		
	$D = 0.05\,\mu m^2/s$	(1, 2, 3)	
Y-MEM receptors	$r^{coll}_{stem} = 5.0\,nm$	(19)	
	$D = 0.05\,\mu m^2/s$	(1, 2, 3)	
	other parameters equal those of Y-SOL receptors		

Table S2. Down-scaling of parameter values for simulated ABM. ABM are down-scaled by factor $s = 0.01$ to obtain computationally feasible system size, *i.e.* the number of molecules (n) and the radius (r) of the cell and the spatial environment were decreased keeping molecule concentrations constant.

model component	original value	down-scaled value
environment	$r = 7.56\,\mu m$	$r = 1.33\,\mu m$
cell	$r = 6\,\mu m$	$r = 0.6\,\mu m$
ligand	$n = 90480$	$n = 904$
receptors	$n = 90480$	$n = 904$

Table S3. Steady state values of complex (C) and receptor (R) concentration observed by fitting the ODE model to dynamics of O-SOL ABM variant.

k_{on}^{micro} $[\mu m^3/s]$	**C** $[1/\mu m^3]$	**R** $[1/\mu m^3]$
10^4	80.442	19.558
5×10^4	90.636	9.364
10^5	93.291	6.709
5×10^5	96.839	3.161
10^6	97.740	2.260
2.5×10^6	98.428	1.572
4×10^6	98.673	1.327
5×10^6	98.779	1.221
6×10^6	98.864	1.136
7.5×10^6	98.907	1.093
10^7	98.996	1.004
1.5×10^7	99.068	0.932
2.5×10^7	99.132	0.868

Table S4. Steady state values of complex (C) and receptor (R) concentration observed by fitting the ODE model to dynamics of O-MEM ABM variant.

k_{on}^{micro} $[\mu m^3/s]$	**C** $[1/\mu m^3]$	**R** $[1/\mu m^3]$
10^4	72.462	27.538
5×10^4	86.067	13.933
10^5	89.876	10.124
5×10^5	94.947	5.053
10^6	95.991	4.009
2.5×10^6	96.949	3.051
4×10^6	97.185	2.815
5×10^6	97.284	2.716
6×10^6	97.363	2.637
7.5×10^6	97.414	2.586
10^7	97.526	2.474
1.5×10^7	97.596	2.404
2.5×10^7	97.640	2.360

Table S5. Steady state values of complex (C) and receptor (R) concentration observed by fitting the ODE model to dynamics of Y-SOL ABM variant.

k_{on}^{micro} $[\mu m^3/s]$	**C** $[1/\mu m^3]$	**R** $[1/\mu m^3]$
10^4	80.378	19.622
5×10^4	90.589	9.411
10^5	93.172	6.828
5×10^5	96.888	3.112
10^6	97.725	2.275
2.5×10^6	98.469	1.531
4×10^6	98.742	1.258
5×10^6	98.841	1.159
6×10^6	98.900	1.100
7.5×10^6	98.956	1.044
10^7	99.067	0.933
1.5×10^7	99.142	0.858
2.5×10^7	99.220	0.780

Table S6. Steady state values of complex (C) and receptor (R) concentration observed by fitting the ODE model to dynamics of Y-MEM ABM variant.

k_{on}^{micro} $[\mu m^3/s]$	**C** $[1/\mu m^3]$	**R** $[1/\mu m^3]$
10^4	72.279	27.721
5×10^4	85.697	14.303
10^5	89.534	10.466
5×10^5	94.814	5.186
10^6	96.002	3.998
2.5×10^6	97.021	2.979
4×10^6	97.295	2.705
5×10^6	97.392	2.608
6×10^6	97.472	2.528
7.5×10^6	97.550	2.450
10^7	97.647	2.353
1.5×10^7	97.759	2.241
2.5×10^7	97.765	2.235

Table S7. Parameter values a and b in the mapping of microscopic and macroscopic binding rates for different ABM variants.

**model**	**a** $[\mu m^3 s^{-1}]$	**b**$\times 10^6$ $[s^{-1}]$
O-SOL	16.6	8.2
O-MEM	1.8	2.1
Y-SOL	22.3	11.4
Y-MEM	2.1	2.7

Table S8. Parameter values f_s and f_r for different ABM variants. The scaling factors f_s and f_r are calculated from equations (17) and (18) for parameters specific to the considered ABM variant.

**model**	f_s	f_r	$\frac{f_s - f_s^{O-SOL}}{f_s^{O-SOL}}$	$\frac{f_r - f_r^{O-SOL}}{f_r^{O-SOL}}$
O-SOL	1.02	0.79	0	0
O-MEM	0.23	0.21	-0.77	-0.74
Y-SOL	1.37	0.76	0.35	-0.04
Y-MEM	0.31	0.31	-0.69	-0.61

REFERENCES

1 .Treanor B, Depoil D, Gonzalez-Granja A, Barral P, Weber M, Dushek O, et al. The membrane skeleton controls diffusion dynamics and signaling through the B cell receptor. *Immunity* **32** (2010) 187–199.

2 .Liu W, Meckel T, Tolar P, Sohn HW, Pierce SK. Antigen affinity discrimination is an intrinsic function of the B cell receptor. *The Journal of experimental medicine* **207** (2010) 1095–1111.

3 .Favier B, Burroughs NJ, Wedderburn L, Valitutti S. TCR dynamics on the surface of living T cells. *International immunology* **13** (2001) 1525–1532.

4 .Einstein A. Über die von der molekularkinetischen Theorie der Wärme geforderte Bewegung von in ruhenden Flüssigkeiten suspendierten Teilchen. *Macromolecular Symposia* **322** (1905) 549–560.

5 .Tsourkas PK, Baumgarth N, Simon SI, Raychaudhuri S. Mechanisms of B-cell synapse formation predicted by Monte Carlo simulation. *Biophysical journal* **92** (2007) 4196–4208.

6 .Tsourkas PK, Longo ML, Raychaudhuri S. Monte Carlo study of single molecule diffusion can elucidate the mechanism of B cell synapse formation. *Biophysical journal* **95** (2008) 1118–1125.

7 .Tsourkas PK, Raychaudhuri S. Modeling of B cell synapse formation by monte carlo simulation shows that directed transport of receptor molecules is a potential formation mechanism. *Cellular and Molecular Bioengineering* **3** (2010) 256–268.

8 .Tsourkas PK, Somkanya C D, Yu-Yang P, Liu W, Pierce SK, Raychaudhuri S. Formation of BCR oligomers provides a mechanism for B cell affinity discrimination. *Journal of Theoretical Biology* **307** (2012) 174–182.

9 .Schöneberg J, Noé F. ReaDDy–a software for particle-based reaction-diffusion dynamics in crowded cellular environments. *PloS one* **8** (2013) e74261.

10 .Eigen M. Diffusion control in biochemical reactions. *Quantum statistical mechanics in the natural sciences* (Springer US) (1974), 37–61.

11 .Takahashi K, Tanase-Nicola S, ten Wolde PR. Spatio-temporal correlations can drastically change the response of a MAPK pathway. *Proceedings of the National Academy of Sciences of the United States of America* **107** (2010) 2473–2478.

12 .van Zon JS, Morelli MJ, Tnase-Nicola S, ten Wolde PR. Diffusion of transcription factors can drastically enhance the noise in gene expression. *Biophysical journal* **91** (2006) 4350–4367.

13 .Shoup D, Szabo A. Role of diffusion in ligand binding to macromolecules and cell-bound receptors. *Biophysical journal* **40** (1982) 33–9.

14 .Zwanzig R. Diffusion-controlled ligand binding to spheres partially covered by receptors: an effective medium treatment. *Proceedings of the National Academy of Sciences of the United States of America* **87** (1990) 5856–5857.

15 .DeLisi C. The effect of cell size and receptor density on ligand-receptor reaction rate constants. *Molecular Immunology* **18** (1981) 507–511.

16 .Berg HC, Purcell EM. Physics of chemoreception. *Biophysical journal* **20** (1977) 193–219.

17 .Rozenberg G. *Microscopic haematology: a practical guide for the laboratory* (Elsevier Australia) (2011), 250 .

18 .Carrasco YR, Fleire SJ, Cameron T, Dustin ML, Batista FD. LFA-1/ICAM-1 interaction lowers the threshold of B cell activation by facilitating B cell adhesion and synapse formation. *Immunity* **20** (2004) 589–599.

19 .Maity PC, Yang J, Klaesener K, Reth M. The nanoscale organization of the B lymphocyte membrane. *Biochimica et Biophysica Acta - Molecular Cell Research* **1853** (2015) 830–840.

20 .Murphy RM, Slayter H, Schurtenberger P, Chamberlin RA, Colton CK, Yarmush ML. Size and structure of antigen-antibody complexes. Electron microscopy and light scattering studies. *Biophysical journal* **54** (1988) 45–56.

21 .Amzel LM, Poljak RJ. Three-dimensional structure of immunoglobulins. *Annual review of biochemistry* **48** (1979) 961–97.

22 .Pease LF, Elliott JT, Tsai DH, Zachariah MR, Tarlov MJ. Determination of protein aggregation with differential mobility analysis: application to IgG antibody. *Biotechnology and bioengineering* **101** (2008) 1214–22.

23 .Fan Zc, Shan L, Guddat LW, min He X, Gray WR, Raison RL, et al. Three-dimensional structure of an Fv from a human IgM immunoglobulin. *Journal of Molecular Biology* **228** (1992) 188–207.

24 .Czajkowsky DM, Shao Z. The human IgM pentamer is a mushroom-shaped molecule with a flexural bias. *Proceedings of the National Academy of Sciences of the United States of America* **106** (2009) 14960–14965.
